# Case Report: Low-profile covered stents in CHD: the role of Gore Viabahn (VBX) stents

**DOI:** 10.3389/fped.2026.1739273

**Published:** 2026-03-26

**Authors:** Micol Rebonato, Catarina Almeida, Mara Pilati, Enrico Piccinelli, Gianfranco Butera

**Affiliations:** 1 Cardiology, Cardiac Surgery and Heart Lung Transplantation, Bambino Gesù Children’s Hospital, IRCCS, Rome, Italy; 2 Cardiology, Hospital San Joao, Porto, Portugal

**Keywords:** children, congenital heart disease, Gore Viabahn (VBX) stents, interventional cardiology, vessel stenosis

## Abstract

The Gore Viabahn covered stent, approved for the treatment of peripheral arterial disease in femoral or iliac artery lesions, may offer advantageous features in the field of congenital heart diseases. To date, only a few cases in the literature have reported the off-label use of these stents. Here, we report on four cases in which the use of Gore Viabahn stents was successful across various substrates.

## Introduction

The Gore Viabahn balloon-expandable endoprosthesis (W. L. Gore & Associates) is a flexible, premounted covered stent approved for the treatment of patients with symptomatic peripheral arterial diseases (1). These stents have features that might make them advantageous for the percutaneous treatment of lesions in patients with congenital heart disease. In this case series of four patients, we describe our experience with the Viabahn stent in different clinical settings, thereby contributing scientific knowledge to its off-label use of these scaffolds.

## Case 1

**Figure 1:**
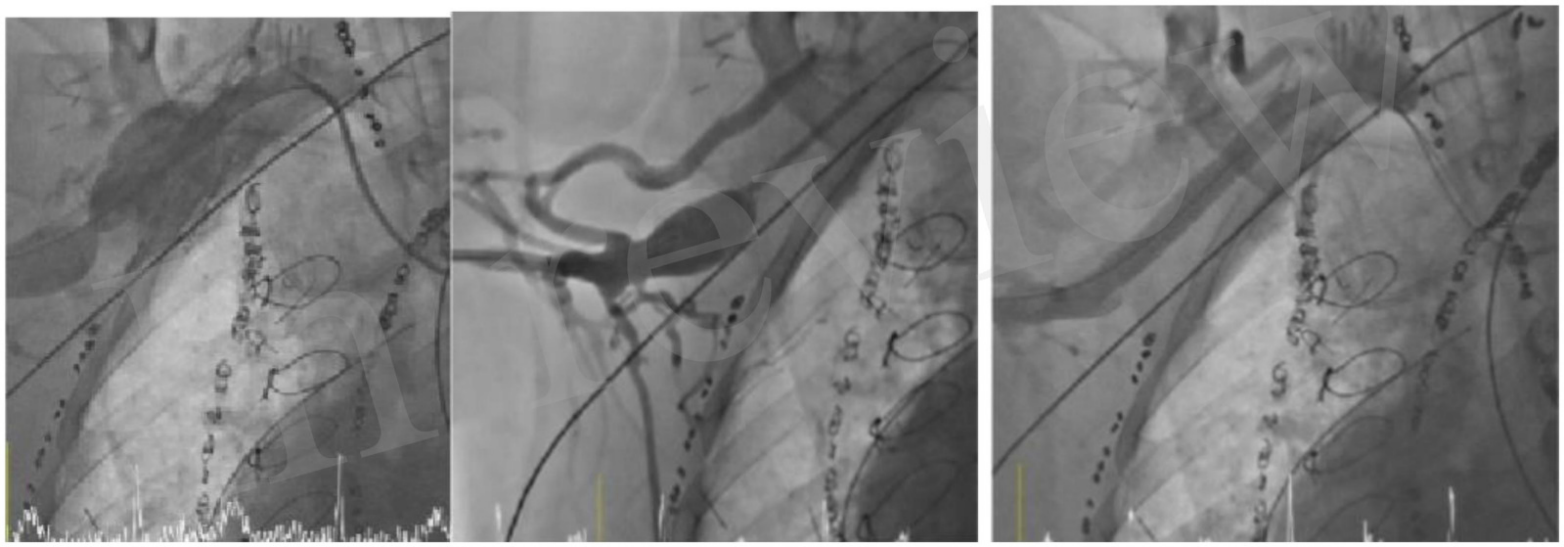
Right subclavian artery angiogram depicting the massive aneurysmal fistula and angiographic occlusion following an 8 × 59-mm Gore Viabahn stent placement.

An 18-year-old male patient, weighing 75 kg, with hypoplastic left heart syndrome had undergone multiple palliated interventions, including a Norwood-Sano and Glenn procedure in the first year of life, followed by creation of a right axillary arteriovenous fistula at 8 years of age because of diffuse right pulmonary arteriovenous malformations. He remained under follow-up at our unit. Due to severe intractable hypoxemia, he underwent a heart transplant at the age of 18. In the postoperative period, a cardiac catheterization was performed to close the right axillary arteriovenous fistula because of right heart failure secondary to AVF-associated volume overload. Percutaneous access was established via the left axillary artery (6 Fr) and right axillary vein (7 Fr). Selective angiography of the right subclavian artery revealed a proximal diameter of 7 mm tapering to 6.5 mm distally, with a large aneurismal arteriovenous fistula and significant ectasia of the superior vena cava. The decision was made to exclude the AV fistula with a covered stent. According to the axillary artery diameter (Rev 2), an 8 × 59-mm Gore Viabahn stent was chosen to seal the ostium of the fistula. After implantation, there was a residual leak at the distal end of the graft that was finally closed with a 14-mm vascular plug, with complete exclusion of the flow through the fistula (Figure 1).

A right arm ultrasound performed 1 week following the procedure showed no residual flow into the aneurysm and preserved good flow through the stented axillary artery. The postoperative course was complicated by severe hypoxemia due to the presence of multiple pulmonary arteriovenous fistulas, which required prolonged mechanical ventilation and recurrent pulmonary superinfections. The patient ultimately progressed to a terminal phase of cardiogenic shock and sudden death.

## Case 2

A 22-month-old boy, weighing 7 kg, was born with a double-outlet right ventricle with pulmonary atresia, interventricular septal defect, discontinuous left pulmonary artery from the arterial duct, major aortopulmonary collateral arteries, mitral stenosis, and a persistent left superior vena cava to coronary sinus. He underwent several procedures: stent implantation in the arterial duct at 16 days of life, balloon atrioseptostomy due to his mitral stenosis at 5 months, and Major Aorto-Pulmonary Collateral Arteries (MAPCA) unifocalization with placement of a central Lacks shunt and atrioseptostomy at 21 months of age. Eight days after surgery, the patient underwent cardiac catheterization because of prolonged and massive fluid drainage from the mediastinal drains. Plasma leak from a Gore-Tex shunt can occur as complication, likely due to an inability of the surrounding tissue to seal the graft. Angiography showed a 6-mm central shunt with proximal tortuosity, which was accessed in an anterograde way with an 8-Fr long sheath (Mullins) advance over a 0.035″ guidewire positioned in the left pulmonary artery. Mean arterial pulmonary pressure was about 16 mmHg and oxygen saturation 75% (Rev 2). A covered Gore Viabahn 8 × 39-mm stent was implanted in order to reduce the permeability of the Gore-Tex shunt, thus reducing the plasma leak but maintaining good flow inside. To ensure stability, we oversized the stent by approximately 2 mm relative to the shunt's nominal diameter (Rev 2).

**Figure 2:**
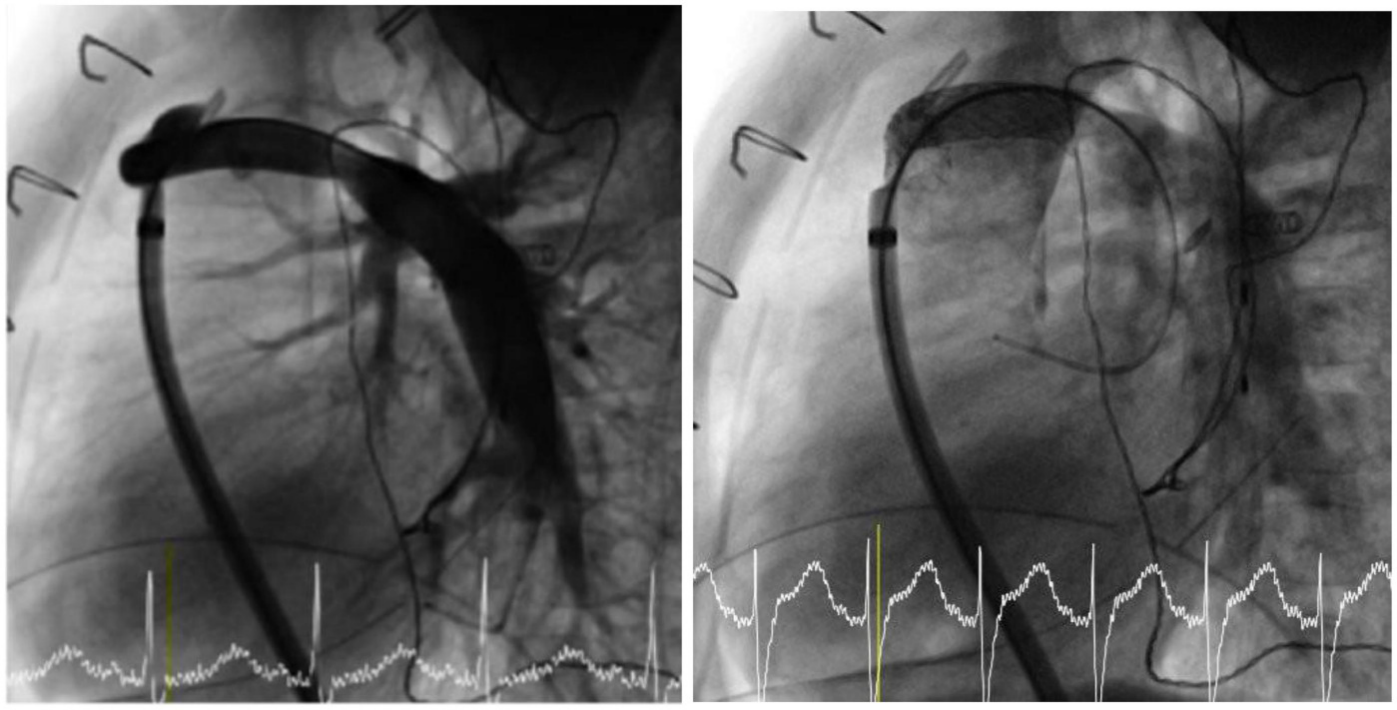
A covered Gore Viabahn 8 × 39-mm stent was implanted in the central shunt with total coverage of the aortic portion and preserved pulmonary branches distal flow.

Poststent angiography confirmed complete coverage of the aortic portion and preserved pulmonary branch distal flow (Figure 2). Two days after catheterization, the mediastinal and pleural drains were removed. The patient demonstrated clinical stability, with oxygen saturation improving to 86%, and was discharged 6 days after the last drain removal. At 24 months of follow-up, no recurrence of fluid collection around the graft was observed, and the boy remained asymptomatic.

## Case 3

A 14-year-old girl, weighing 64 kg, was born with a hypoplastic aortic arch and isthmic coarctation. She underwent four surgical procedures in the first year of life for aortic arch enlargement and coarctation repair. However, follow-up revealed severe aortic recoarctation with multiple obstruction sites and moderate reduction of transverse arch diameter. The girl presented with high blood pressure values despite dual antihypertensive therapy.

**Figure 3:**
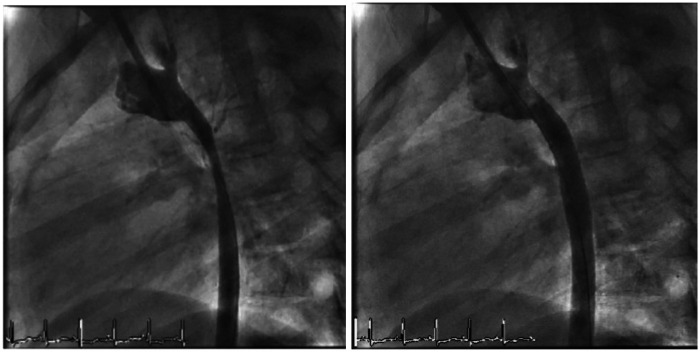
Severe aortic re-coarctation treated with an 11 × 59-mm Gore Viabahn stent with optimal angiographic result.

During catheterization, the aorta was surgically accessed via the right carotid artery cut-down (10 Fr) because of chronic occlusion of both femoral arteries. An initial 100 mmHg peak-to-peak systolic gradient was measured from the ascending to descending aorta. Angiography revealed the following measures: 20 mm ascending aorta, 5 mm isthmic stenosis with severe hypoplasia from the isthmus to the thoracic aorta (6 mm diameter), and 11 mm at diaphragmatic level. Given the patient’s history of multiple previous surgical reinterventions and the risk of potential wall dissection, we decided to treat the residual lesion with a covered stent. An 11 × 59-mm Gore Viabahn stent was advanced through a short 10 Fr sheath and deployed directly in order to cover the entire hypoplastic region. The proximal portion was postdilated with a 12 × 20-mm Atlas balloon. At the end of the procedure, the final aortic peak-to-peak systolic gradient was 15 mmHg, with an optimal angiographic result (Figure 3). The postoperative course went uneventful and the girl was discharged 3 days later. At the 8-month follow-up, her blood pressure remained controlled with a single antihypertensive therapy.

## Case 4

A 3-year-old girl, weighing 10 kg, with Tetralogy of Fallot, absent pulmonary valve, and discontinuous pulmonary arteries initially underwent complete surgical repair at 5 days of life. After 1 year of age, because of severe left pulmonary artery stenosis due to extrinsic compression, she underwent another surgical pulmonary artery plasty associated with a Lecompte maneuver. Despite reintervention, no improvement was achieved.

**Figure 4:**
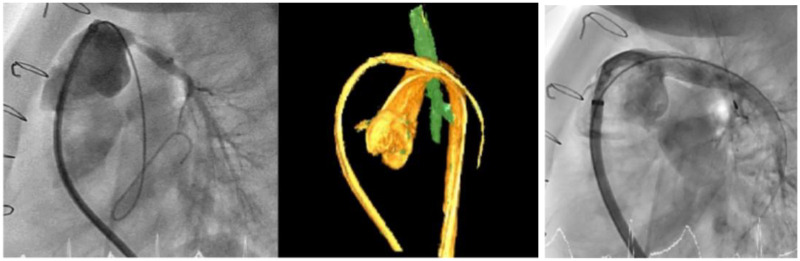
Severe stenosis at the origin of the left pulmonary artery and diffuse hypoplasia of the distal part treated successfully with an 8 × 29-mm Gore Viabahn stent.

Cardiac catheterization was performed in order to treat the lesion, demonstrating severe stenosis at the origin of the left pulmonary artery (1 mm diameter) and diffuse hypoplasia of the distal part (3.5 mm) with a mean pressure of 6 mmHg. Using 3D rotational angiography, an 8 × 20-mm Mustang balloon was utilized to test both pulmonary artery distensibility and its distance from the left bronchus to exclude potential bronchial compression. Given the close proximity between the left pulmonary artery (LPA) and the aorta, and to minimize the risk of aortic erosion, an 8 × 29-mm Gore Viabahn stent (8 mm was the maximal vessel distension diameter REV 2) was finally implanted into the left pulmonary branch with optimal result (Figure 4).

At angiographic control, the stent demonstrated a homogeneous caliber without need for postdilation. The mean distal pulmonary pressure was 12 mmHg with good pulsatility (Rev 2).

The child was discharged the following day on aspirin therapy, with oxygen saturation of 99%. At the 1-year follow-up, echocardiography confirmed the left pulmonary artery of appropriate diameter with preserved flow and normal pressure in the right ventricle.

## Discussion

This case series reports the successful off-label application of the Gore Viabahn stent in patients with complex congenital vascular lesions, despite the device not being originally designed for this indication.

The Gore Viabahn stent consists of individual stainless steel ring elements that support a heparinized Gore-Tex tube (W. L. Gore & Associates) capable of segmental flaring with maximal flexibility and great radial strength. These covered scaffolds offer promising characteristics even in small vessels because of the relatively low profile; in fact, these stents can be implanted using sheaths ranging from 6 Fr up to a maximum of 8 Fr.

The 5–7-mm stents have a maximal postdilated diameter of 8–11 mm, whereas the 8–11-mm stents can be dilated from 13 to 16 mm, allowing for wider applicability in the pediatric setting, especially in growing children, where the ability to postdilate allows for adjustments as the child grows.

With the risk of arterial tear with standard balloon-expandable stents, covered stents—such as the VBX endoprosthesis—may provide protection from vascular lesions and are preferred in high-risk situations, such as vessel wall dissection. The standard covered stents used conventionally in the laboratory—such as the CP, Optimus, or BeGraft stent—are often more rigid and may be better suited for adult patients. While they offer excellent and reliable performance in certain cases, they may not be ideal in small patients due to their higher profile. As a matter of fact, stents of a similar size require at least a 9-Fr sheath and in some cases even larger (e.g., an 8-zig covered Cheatham platinum stent requires a 12-Fr sheath) (2). The Viabahn VBX stent foreshortens by a median value of 19.3% (range 4.4%–36.1%) when expanded toward its maximum diameter, similar to other balloon-expandable covered stents (3).

The Gore Viabahn stent's features, such as flexibility, radial strength, and protection against vascular trauma, have shown promising benefits in the context of congenital heart disease. Beyond their general mechanical properties, the specific characteristics of the Gore Viabahn VBX stent played a decisive role in device selection across the clinical scenarios included in this series. In smaller patients, the low delivery profile of the VBX stent was a key determinant, as it allowed safe implantation through 6–8 Fr sheaths, thereby minimizing vascular access trauma and reducing the risk of access site complications. This consideration was particularly relevant in younger patients and those with limited vascular caliber, in whom the use of higher-profile covered stents—such as CP, Optimus, or BeGraft devices—would have necessitated larger introducer sheaths and potentially increased procedural risk. In addition, the segmented ring design and inherent flexibility of the VBX stent favored its use in complex or tortuous anatomies, where rigid covered stents may exert excessive focal stress on the vessel wall. This conformability was especially advantageous in lesions adjacent to surgical anastomoses, patch material, or previously manipulated vessels, where the risk of dissection or rupture is heightened. In such settings, the combination of controlled radial force and immediate coverage provided by the VBX stent offered both effective lesion treatment and protection against acute vascular injury (REV 2).

To date, the literature includes a case series by Cole et al. (6), detailing the use of Gore Viabahn VBX covered stents in a small pediatric cohort ranging from a 4.2-kg newborn to a full-grown adult. Indications included closure of an AV fistula, stenting a vertical vein in obstructed supracardiac total anomalous pulmonary venous return, and treatment of severe interposition graft stenosis in type B interrupted aortic arch. They demonstrated that the Gore Viabahn VBX stent provides effective relief of vascular stenoses with good short-term vessel patency. No major complications were reported, and the stents remained patent at follow-up, suggesting promising clinical utility in this population. In addition, two single case reports describe VBX usage in pediatric patients, one for aortic coarctation in a 7-year-old boy and another in a 3-month-old infant with severe left pulmonary artery stenosis in the setting of Tetralogy of Fallot with pulmonary atresia and an RVOT pseudoaneurysm (4, 5).

In our series, we describe four patients aged 22 months to 18 years with severe congenital heart disease and complex vascular anatomies who were successfully treated with Gore Viabahn stents (from 8 × 29 mm to 11 × 59 mm) in a variety of vascular lesions. No procedural complications occurred. At a mean follow-up of 18 months, three patients out of four remained free of symptoms. Ultrasound examination showed that the stents remained patent with preserved lumen and flow, and no signs of restenosis.

Further studies and larger series are needed to determine long-term results, including stent durability, restenosis, and postdilation properties.

## Data Availability

The original contributions presented in the study are included in the article/Supplementary Material, further inquiries can be directed to the corresponding author.
